# Dr. Giehrl’s Case of Spontaneous Cure of Cataract

**Published:** 1844-09-21

**Authors:** 


### DR. GIEHRL'S CASE OF SPONTANEOUS CURE OF CATARACT.

A stone-breaker, who had suffered from cataract of the right eye from his youth, had the misfortune, whilst pursuing his occupation, to have his left eye struck by a splinter, which produced a violent concussion of the eye, and gave rise to inflammation and loss of vision. The man applied to Dr. G., who, on examining the eye, found, along with considerable inflammation, a completely formed cataract. He combated the former symptom by antiphlogistic remedies, and advised the patient, as soon as all irritation in the left eye should have disappeared, to have the cataract removed from the right one, in which the power of vision had been lost at an early period. On this the patient applied elsewhere for advice, and consulted Dr. S. in A.

In order to examine the eye more minutely, the latter dropped into it a solution of the extract of belladonna; in consequence of this the pupil dilated largely; at the same time, the opaque lens fell into the anterior chamber; and vision was immediately restored. The lens became absorbed by degrees, and the patient was cured.—*Ibid. from Allgemeine Zeitung für Chirurgia.*

